# Inconsistency in prevalence of hypertension based on self-reports and use of standard tests: Implications for large scale surveys

**DOI:** 10.1016/j.ssmph.2022.101255

**Published:** 2022-10-03

**Authors:** Shri Kant Singh, Santosh Kumar Sharma, Sanjay K. Mohanty, Rakesh Mishra, Akash Porwal, Bal Kishan Gulati

**Affiliations:** a Department of Survey Research & Data Analytics, International Institute for Population Sciences, Mumbai, India; b International Institute for Population Sciences, Mumbai, India; c Department of Population & Development, International Institute for Population Sciences, Mumbai, India; d Monitoring and Evaluation Consultant, UNICEF, India; e Population Council, India; f National Institute of Medical Statistics, Indian Council of Medical Research, India

**Keywords:** Hypertension, Specificity, Sensitivity, False-positive errors, False-negative errors, India

## Abstract

**Objective:**

Biomarkers are increasingly integrated into population-based surveys to provide reliable estimates of the prevalence of specific diseases. The Demographic and Health Surveys have recently incorporated blood pressure measurements; however, little is known about the extent of agreement between measured and reported levels of hypertension in India. The objective of this study was to examine the extent of agreement between self-reported hypertension and the results of standard blood pressure measurements, as well as to explore the risk groups and factors associated with inconsistencies in self-reported and biomedically measured hypertension.

**Methods:**

Reliability measures such as sensitivity, specificity, and kappa statistics were used to examine inconsistencies in self-reported and biomedically measured hypertension in the National Family Health Survey-4 data. Multilevel logistic models were adopted to analyse the respondent characteristics related to both false-positive and false-negative responses in the survey.

**Results:**

Compared to biomedically measured hypertension, self-reported hypertension was inconsistent and disproportionate at disaggregated levels in India. While self-reports severely underestimated hypertension among men aged 15–54 years and women aged 35–49 years, it overestimated hypertension among women below the age of 35 years. The inconsistency in self-reported and biomedically examined hypertension had deviations from a sex standpoint. Women aged <35 years reported a false-positive prevalence of hypertension. False-negative responses were elucidated among women aged ≥35 years and men aged 15–54 years. The likelihood of false-positive responses was higher among pregnant and obese respondents, and those who consumed alcohol.

**Conclusion:**

The significant deviance of self-reporting of hypertension from the prevalence derived based on standard tests further indicates the need for adopting standard tests in all emerging future large-scale surveys. A back-check survey is recommended to understand and differentiate the excessive false-positive reporting of hypertension among women aged 15–35 years.

## Highlights

• Severely underestimated hypertension among men aged 15–54 and women aged 35–49, overestimated the condition among women below the age of 35.
• Over-reporting of the false-positive prevalence of hypertension in the women below the age of 35.
• False-negative responses were higher among the women aged 35 and above and the men aged 15–54.
• Higher likelihood of the false-positive responses among the pregnant respondents and obese men and women, and the ones consuming alcohol.
• Interventions are required to raise awareness of the necessity of physical examinations and basic health education in India.

## 1 Introduction

Hypertension is a well-known risk factor for cardiovascular disease. It is the leading cause of death worldwide, with 17.8 million deaths reported in 2017 (Xie & Wang, 2020). High-quality estimates of prevalence based on biomedical measurements are needed to monitor cardiovascular disease risks and plan public health prevention and interventions. Owing to the high cost and long-term collection of biomedical data, economists, demographers, and public health professionals have relied heavily on self-reported hypertension to estimate its prevalence and disease burden (Wu et al., 2013). However, recent research has raised questions regarding the reliability of self-reported health status (Murray & Chen, 1992; Newell et al., 1999).

In public health surveys, respondents are often asked about their medical history and current health conditions to determine their risk status and vulnerability to certain diseases (Merkin et al., 2007). Although it is convenient and cost-effective to gather health status data through self-reporting, the quality of the data remains questionable (Iversen, Hannaford, Godden, & Price, 2007; Ungar & Coyte, 1998). Studies have highlighted substantial disagreement between self-reported and medically recorded diabetes, hypertension, pulmonary disease, cerebrovascular disease, and myocardial infarction (Hassey et al., 2001; Whitelaw et al., 1996). Reliable estimates based on biomedical measurements of these diseases are essential for research and the planning and implementation of public health policies aimed at preventing various cardiovascular diseases (Ning et al., 2016; Puri, 2020). However, in the absence of clinically tested data, most studies were based on the self-reported prevalence of disease. It is important to mention that self-health reporting imposes serious challenges in developing countries, especially where socioeconomic vulnerability is pronounced. In addition, the accuracy of self-reported morbidity is contingent upon participants’ awareness, recall ability, and willingness to report (Iversen et al., 2007; Wolinsky et al., 2014). This leads to a significant gap between the reported and actual figures, often resulting in a huge data quality issue. To the best of our knowledge, this is the first attempt to address such data quality issues regarding hypertension in any national-level survey in India.

Biomedical measurement of diseases is always considered ‘the gold standard’ in concordance studies, that is, discrepancies between self-reports and claims are interpreted as misreporting by self-reports (Wolinsky et al., 2007, 2014; Zuvekas & Olin, 2009). The validity of self-reports is questionable when investigating specific diseases such as diabetes and hypertension (Margolis et al., 2008). The degree of under-ascertainment of hypertension cases by self-reporting is relatively less well described, and previous studies have not quantified the performance of self-reported hypertension (Schneider et al., 2012). A small and nascent body of research comparing the self-reported status of certain diseases with the true status based on clinical diagnoses has found significant gaps. A study conducted by Okura, Urban, Mahoney, Jacobsen, and Rodeheffer (2004) articulated that the agreement between self-reported and medical records was substantial (kappa 0.71–0.80) for diabetes and hypertension. These validation exercises predominantly used data from high-income countries (Onur & Velamuri, 2018; Ning et al., 2016) and reported a moderate agreement between self-reported prevalence and results based on standard tests for hypertension and diabetes through the China Health and Retirement Longitudinal Study. Johnston et al. (2009) noted an average of 28% under-reporting and an attenuation bias of 68% between self-reported and clinically tested hypertension in a health survey conducted in England. Dyrstad et al. (2014) found that different patient characteristics had an impact on the agreement between self-reported and tested measures.

Maintaining adequate data quality is crucial for better monitoring and evaluation of the existing policies and programs. Arguably, improved data quality ensures reliability of the estimates, which in turn helps in the appropriate assessment of various programmatic interventions at the granular level. Despite the vast body of literature, various health indicators in large-scale surveys rely on self-reported estimates. These estimates are affected by a variety of biases at both the interviewer and respondent levels (Wolinsky et al., 2007, 2014). Thus, validation of reporting errors becomes an indispensable approach towards ensuring data quality in countries where a significant share of the population is socioeconomically vulnerable to the knowledge of health risks (Johnston et al., 2009). In fact, lack of awareness and misinformation regarding health conditions cause deviation from an adequate response, severely impacting data quality and the policies formed based on such estimates among countries with massive population sizes. As a remedy, the Indian Demographic and Health Survey has included biomarker tools to capture the exact health status of men and women and facilitate specifics on various risk stratifications. At the same time, it also provides an opportunity to examine consistency in self-reported health status at various disaggregated levels. There is inadequate empirical evidence of inconsistencies between biomarker tests and self-reports in developing countries such as India, and little is known about the causes and specifics of these variations.

Thus, it is imperative to examine the concordance or discordance between self-reported and measured diagnostic data on health status for informed policy suggestions or decisions regarding data quality. Considering the above facts, this study aimed to understand and differentiate the gradients of inconsistencies in hypertension reporting, which is a recognised health challenge in India. The two broader objectives are: i) comprehensive evaluation of data quality on hypertension by examining the disagreement between self-reported disease and biomedical measurements, and ii) exploring socioeconomic factors leading to such heterogeneity.

## 2 Materials and methods

### 2.1 Data

This study used survey data from the India National Family Health Survey (NFHS)-4, conducted from 2015 to 2016. The NFHS collects and disseminates information on important aspects of maternal, child, and adult health indicators. A special feature of the NFHS-4 is testing of the adult population for blood pressure within the biomarker components. In the biomarker schedule, self-reported information was collected from both men and women using a series of questions such as “*Were you told on two or more different occasions by a doctor or other health professional that you had hypertension or high blood pressure?*”, “*Before this survey, has your blood pressure ever been checked?”,* and “*Are you currently taking prescribed medication to lower your blood pressure?*”. However, in the Clinical, Anthropometric, and Biochemical (CAB) survey contained within the NFHS-4, blood pressure was measured for all eligible women aged 15–49 and eligible men aged 15–54, using an Omron blood pressure monitor, to determine the prevalence of hypertension. Blood pressure measurements for each respondent were taken three times with an interval of 5 min between readings. We averaged the last two readings after excluding the first reading to avoid white-coat hypertension. Respondents whose average systolic blood pressure was ≥140 mmHg or average diastolic blood pressure was ≥90 mmHg were considered to have elevated blood pressure readings (IIPS and ICF 2017). These data were then collated to develop an indicator of hypertension based on the recommendations of the World Health Organization (WHO) (Organization, 2013).

### 2.2 Dependent variable

As the dependent variable, the study used hypertension based on self-reports and measured it using standard tests among women (15–49 years) and men (15–54 years). In the NFHS-4 (2015–16), according to the WHO guidelines (WHO, 2011), an individual is classified as having hypertension if the systolic blood pressure ≥140 mm Hg, or diastolic blood pressure ≥90 mm Hg, and/or is currently using antihypertensive medication. Based on the reported and biologically tested hypertension results, studies have classified responses into four exclusive categories. In the first category, self-reported hypertension correctly matches the medically tested results. This is known as the ‘gold standard’ (Huerta et al., 2009) and such responses are labelled as true-positive. The second category is recognised as true-negative, where the absence of the tested hypertension is correctly reported. The third category of responses is identified as false-negative, where respondents falsely report themselves as hypertensive in the survey, without being medically reported for the condition. Such cases often overreport the actual prevalence of ailments and are policy concerns for any country. The fourth category is identified as false-positive, which covers all non-responses of self-reported hypertension that are tested positive using standard tests (Huerta et al., 2009). The dependent variables in the study were false-positive (FP) and false-negative (FN) responses to hypertension.

### 2.3 Independent variable

Socioeconomic and demographic characteristics, such as the age of the respondents (15–24, 25–34, or 35–49 years), educational attainment (no education, primary, secondary, or higher education), place of residence (urban or rural), currently pregnant (yes or no), body mass index (normal, underweight, obese, or not known), religion (Hindu, Muslim, or others), social caste group (SC/ST, OBC, general, or others), wealth quintile (poorest, poorer, middle, richer, or richest), and region (north, north-east, central, eastern, western, or south) were used as independent variables in this study. Besides these variables, substance abuse in the form of alcohol and tobacco consumption was also considered as explanatory variables.

### 2.4 Methodology

To assess the difference in prevalence estimates based on the data collection method used, the prevalence of hypertension was calculated according to self-reported information as well as according to the results of biomedical measurements obtained from the survey. The degree of underestimation or overestimation was computed as follows:

$$\mu =\frac{Biomedical\;test\;–Self\;reported\;}{Biomedical\;test}*100$$

Sensitivity, specificity, and kappa statistics were used to assess the accuracy of the self-reported data. The results of biomedical measurements were treated as the ‘gold standard’ for the diagnosis of hypertension. Sensitivity was defined as the percentage of respondents who self-reported hypertension among those diagnosed with hypertension. Specificity was defined as the percentage of individuals who self-reported not having hypertension, among those with ‘normal’ or ‘healthy’ biomedical measurements. A 95% confidence interval (CI) was calculated for sensitivity and specificity estimates across different subgroups. Cohen’s kappa (κ) coefficients were calculated to estimate the overall agreement between the self-reports and biomedical tests. In terms of the κ value, the level of agreement was considered slight (≤0.20), fair (0.21–0.40), moderate (0.41–0.60), substantial (0.61–0.80), or almost perfect (≥0.81) (Cohen, 1960).

Furthermore, several random intercept multilevel logistic regressions were estimated to determine the effect of nested-level cofactors on the likelihood of FP and FN responses to hypertension. The application of multilevel modelling was justified by the hierarchical structure of the survey, where women and men were nested within households and the households were nested within PSUs. Based on the descriptive observations, in the first model, women’s FP reporting of hypertension was analysed against background characteristics, household level, and PSU level factors. In the second and third models, the determinants of FN reporting on hypertension were explored using the same set of predictors. The underlined model was developed by Goldstein, Browne, and Rasbash (2002).

$$\log\left(\frac{p_{ijk}}{1-p_{ijk}}\right)=\beta_{o}+{\beta_{1}x}_{ijk}+\ldots +{\beta_{m}x}_{ijk}+u_{1jk}x_{ijk}+v_{0k}+u_{0jk}+e_{ijk}$$

where *i*, *j,* and *k* are the levels included in the analysis. “*i*” refers to first level inferring to the sex-based variations, whereas “*j*” indicates the household level and “*k*” refers to the PSU (community) level variations. In addition, $p_{ijk}$ is the probability of the *i*th person of the *j*th household and the *k*th PSU reporting FP or FN responses, where FP and FN are binary variables with $y_{ijk}∼Bernoulli\left(p_{ijk}\right)$. Further, $\beta_{i}^{\prime }s$ indicates the regression coefficients corresponding to each explanatory variable in the random intercept model. Additionally, $u_{1jk}$refers to the random effect of the explanatory variable at Level 1 and $x_{ijk}^{\prime }s$ at the second level. $\nu_{0k}$ on the other hand, shows a random effect at the household level, presenting a deviation from the mean responses at the household level. $u_{ojk}$ describes a random departure from the mean effects at the community level. $e_{ijk}$ is the error term bearing the randomness of all levels and is assumed to be independently and identically normally distributed. Furthermore, the error is assumed to be uncorrelated at all three levels. The intraclass correlation coefficient for three-level logistic model similarity in the FP and FN reporting at the household level within the same PSUs is given as $ICC=\sigma_{u}^{2}/\left(\sigma_{u}^{2}+\sigma_{v}^{2}+3.29\right)$, where $\sigma_{u}^{2}$ indicates the variance at the household level and $\sigma_{v}^{2}$ indicates the variance at the PSU level.

## 3 Results

### 3.1 Discrepancies in the self-reported and biomedical measurement of hypertension

**Fig. 1:**
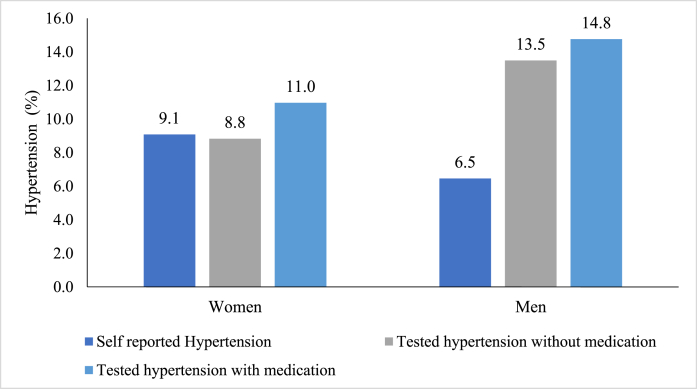
Prevalence of self-reported and measured hypertension among women (15–49 years) and men (15–54 years) in India, NFHS-4, 2015-16.

The prevalence of hypertension among women aged 15–49 years was 11.0% based on standard tests with medication, and 9.1% based on self-reported data. This leads to a 17.2% mismatch between these two measurement methods, which may be due to undiagnosed hypertension. Similarly, the prevalence of hypertension among men aged 15–54 years was 14.8% based on standard tests and 6.5% based on self-reports, providing a substantial mismatch of 56%. An evident gap in the prevalence of self-reported and medically examined hypertension was noted between men and women who were not taking any medication at the time of the survey. The reported differences were 2.8% among women and 52% among men during 2015–16 (Fig. 1).

The discrepancy between self-reported and measured hypertension was dependent on the respondent’s characteristics, the nature of the disease, and current health status. In addition, awareness of ailments and recall ability disproportionality impacted the pattern of self-reporting. Findings from the study indicated a notable discrepancy in the pattern of self-reported and measured hypertension (with and without medication) according to the respondent’s characteristics (Supplementary Table S1). Specifically, it was intriguing to note that the prevalence of self-reported and biomedically diagnosed hypertension for women and men differed across different age groups. The results showed distinct patterns of hypertension reporting for women aged below and above 35 years.

**Fig. 2:**
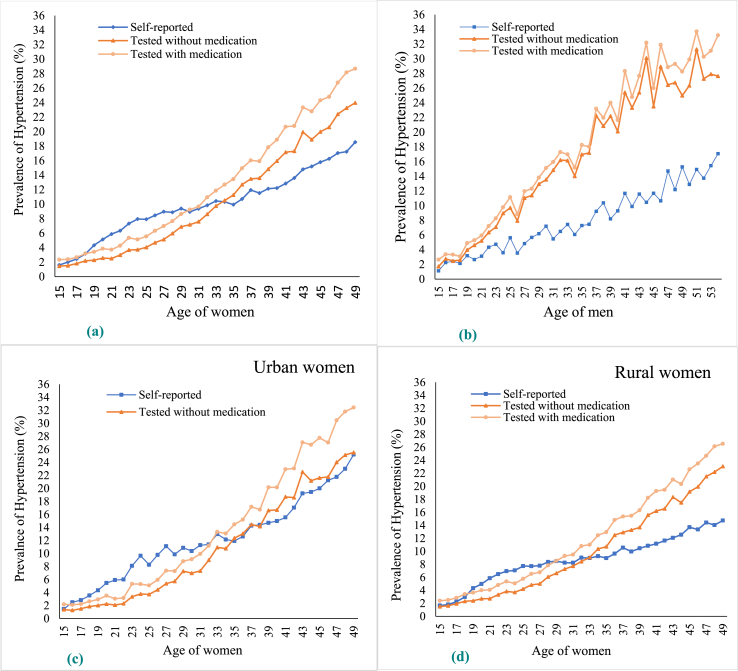
Age pattern in prevalence of self-reported and measured hypertension in India, 2015-16. Note: Fig. 2(a), (c), and (d) are based on all women aged 15–49 years and Fig. 2(b) is based on men aged 15–54 years.

Conspicuously, women aged <35 years over-reported hypertension cases compared to the gold standard, while women aged >35 years under-reported it (Fig. 2a). In contrast, self-reported and biomedically tested hypertension systematically increased among men aged 15–54 years. However, it should be noted that the gap between the two widened with age (Fig. 2b). Notably, the biomedically tested level of hypertension increased with age in both men and women. Similar findings were observed in tested responses in men and women who reported taking medication or not taking medication at the time of the survey. A rural-urban disaggregated analysis of women across ages revealed differences in the patterns of reported and tested hypertension. In urban areas, women aged 16–33 years tended to over self-report hypertension, while in rural areas, over self-reporting was more prevalent among women aged 19–29 years. This crossover between self-reported and tested hypertension in women is quite puzzling and a unique observation of the study. Several studies have highlighted self-underreporting in hypertension in different settings (Ning et al., 2016; Okura et al., 2004; Onur, 2018; Schneider et al., 2012). However, a similar assessment is lacking in India. Moreover, false-positive hypertension among women aged <35 years was a relatively unique observation of this study. Therefore, it is imperative to explore the causes of both self-overreporting and self-underreporting of hypertension in India, especially at disaggregated levels.

### 3.2 Reliability assessment of self-reported and biomedical measurement of hypertension

A significant proportion of men and women correctly identified hypertension at the time of the survey. Table 1 demonstrates the extent of agreement between self-reported and biomedically tested hypertension using reliability statistics such as sensitivity and specificity. The overall sensitivity and specificity of self-reported hypertension among women aged 15–49 years were 41% and 92%, respectively, according to the medically tested hypertension data. This indicated that two of every five women aged 15–49 years correctly identified hypertension, whereas 92% correctly rejected it. Similarly, for hypertension without medication, the sensitivity and specificity among women were 24% and 92%, respectively. However, in men, the sensitivity and specificity remained the same in both with and without medication cases. The sensitivity and specificity of self-reported hypertension among men were 50% and 86%, respectively (Table 1).

**Table 1:** Sensitivity and specificity among women (15–49) and men (15–54), NFHS-4, 2015-16.

	Hypertension with Medication			Hypertension without Medication	
Hypertension	Women	Men		Women	Men
Sensitivity	41.1(40.7–41.5)		50.4(49.3–51.6)	24.4(24.1–24.7)	36.7(35.6–37.8)
Specificity	91.5(91.5–91.6)	85.8(85.6–86.0)		92.2(92.1–92.2)	86.3(86.1–86.5)
ROC area ((Sens.+ Spec.)/2)	66.3(66.1–66.5)	68.1(67.5–68.8)		58.3(58.1–58.5)	62.0(60.8–62.2)

Note: Estimated form NFHS-4, 2015-16.

The overall κ coefficient (kappa statistics) was 0.29 and 0.20 among women and men, respectively, which indicated a fair agreement between self-reported and biomedically tested hypertension. To varying degrees, the differences in κ coefficients between the subgroups were statistically significant (Table 2). Analysis of sensitivity and specificity by the age of the respondents showed that sensitivity (55%) was higher and specificity was lower (84%) among women aged ≥35 years than in those in the younger age groups. Women with higher education had the lowest sensitivity (32%) and highest specificity (94%) for hypertension, which meant that about 68% of women with higher education did not know they had hypertension and 6% falsely thought they had hypertension. Men with higher education had higher sensitivity (50%) and lower specificity (85%), indicating that over 50% of men with higher education did not know they had hypertension, and 15% of men falsely thought they had hypertension.

**Table 2:** Sensitivity, specificity and agreement of self-reported hypertension compared with biomedical data among men and women, NFHS-4, 2015-16.

	Women			Men		
Background Characteristics	**Sensitivity % (95% CI)**	**Specificity % (95% CI)**	**κ (95% CI)**	**Sensitivity % (95% CI)**	**Specificity % (95% CI)**	**κ (95% CI)**
**Age**						
15–24	20.4(19.7–21.2)	96.8(96.7–96.8)	0.178 (0.175–0.182)	22.1(19.4–24.9)	94.4(94.2–94.7)	0.102 (0.095–0.109)
25–34	29.4(28.7–30.0)	92.8(92.7–92.9)	0.218 (0.215–0.222)	38.1(35.9–40.4)	86.9(86.5–97.3)	0.150 (0.142–0.158)
35–49	54.8(54.2–55.3)	84.4(84.2–84.5)	0.316 (0.313–0.320)	57.6(56.0–59.3)	78.6(78.1–79.0)	0.215 (0.207–0.223)
**Education**						
No education	46.9(46.1–47.6)	88.6(88.5–88.8)	0.271 (0.267–0.274)	47.4(43.5–51.2)	85.6(84.9–86.2)	0.171 (0.163–0.178)
Primary	46.0(44.9–47.0)	90.0(89.8–90.2)	0.304 (0.300–0.307)	45.5(41.8–49.2)	85.6(85.0–86.2)	0.170 (0.163–0.178)
Secondary	38.6(38.0–39.1)	93.1(93.0–93.2)	0.300 (0.296–0.303)	45.8(44.1–47.4)	87.6(87.4–87.9)	0.200 (0.192–0.208)
Higher	32.0(30.9–33.1)	93.9(93.7–94.1)	0.270 (0.267–0.274)	50.0(47.3–52.7)	85.0(84.4–85.5)	0.231 (0.223–0.239)
**Current Pregnancy**						
No education	41.9(41.5–42.3)	91.3(91.2–91.3)	0.290 (0.286–0.293)	–	–	–
Yes	22.2(20.6–23.9)	96.6(96.4–96.8)	0.230 (0.226–0.233)	–	–	–
**Body mass index**						
Normal	35.9(35.4–36.5)	92.2(92.1–92.3)	0.247 (0.244–0.251)	43.6(42.1–45.2)	86.9(86.7–87.2)	0.181 (0.173–0.188)
Underweight	28.5(27.5–29.6)	95.1(95.0–95.2)	0.213 (0.210–0.217)	33.0(29.4–36.8)	93.6(93.2–93.9)	0.163 (0.156–0.170)
Obesity	55.2(54.4–55.9)	82.5(82.2–82.7)	0.326 (0.322–0.329)	65.3(63.4–67.1)	71.9(71.2–72.6)	0.234 (0.226–0.241)
Not known	24.4(22.9–25.9)	96.0(95.8–96.2)	0.236 (0.233–0.239)	56.7(37.4–74.5)	80.2(74.8–85.0)	0.239 (0.232–0.247)
**Place of residence**						
Urban	42.0(41.4–42.7)	91.3(91.1–91.4)	0.314 (0.310–0.317)	49.1(47.1–51.1)	85.2(84.4–85.6)	0.219 (0.211–0.227)
Rural	40.5(40.1–41.0)	91.6(91.6–91.7)	0.274 (0.271–0.278)	45.4(43.8–46.9)	87.4(87.1–87.6)	0.187 (0.179–0.195)
**Religion**						
Hindu	38.5(38.0–39.0)	91.9(91.8–92.0)	0.270 (0.266–0.273)	45.2(43.8–46.7)	86.9(86.6–87.1)	0.188 (0.180–0.196)
Muslim	46.5(45.5–47.5)	91.1(90.9–91.3)	0.340 (0.336–0.343)	45.6(42.3–48.9)	88.8(88.3–89.4)	0.227 (0.219–0.236)
Others	47.5(46.6–48.7)	89.5(89.3–89.8)	0.317 (0.314–0.321)	55.6(52.3–58.8)	83.0(82.3–83.7)	0.228 (0.219–0.236)
**Caste**						
SC/ST	40.6(39.9–41.3)	91.2(91.1–91.3)	0.264 (0.260–0.267)	47.9(45.7–50.1)	86.2(85.8–86.6)	0.186 (0.178–0.194)
OBC	36.8(36.2–37.4)	92.2(92.1–92.3)	0.270 (0.267–0.273)	42.2(40.3–44.2)	87.8(87.4–88.1)	0.192 (0.184–0.200)
General	46.1(45.2–46.9)	91.2(91.0–91.3)	0.334 (0.330–0.337)	51.6(49.0–54.3)	85.3(84.8–85.8)	0.220 (0.212–0.228)
Others	53.4(51.7–55.1)	89.6(89.3–90.0)	0.367 (0.363–0.370)	54.3(48.8–59.6)	87.6(86.6–88.4)	0.249 (0.241–0.257)
**Wealth quintile**						
Poorest	36.7(35.5–37.9)	91.9(91.8–92.1)	0.199 (0.195–0.202)	36.7(32.7–40.9)	89.8(89.3–90.3)	0.126 (0.119–0.133)
Poorer	40.6(39.7–41.6)	91.8(91.7–91.9)	0.265 (0.261–0.268)	41.7(38.6–45.0)	88.5(88.1–89.0)	0.161 (0.154–0.169)
Middle	39.9(39.1–40.8)	91.7(91.5–91.8)	0.286 (0.282–0.289)	45.3(42.7–48.0)	86.8(86.4–87.3)	0.192 (0.185–0.200)
Richer	41.1(40.3–41.9)	91.0(90.8–91.2)	0.302 (0.298–0.305)	48.9(46.4–51.3)	84.9(84.4–85.5)	0.213 (0.205–0.221)
Richest	44.2(43.4–45.0)	91.2(91.0–91.3)	0.341 (0.338–0.345)	52.0(49.6–54.3)	83.7(83.1–84.2)	0.233 (0.225–0.241)
**Alcohol Consumption**						
No	40.8(40.4–41.2)	91.7(91.6–91.8)	0.290 (0.286–0.293)	45.4(43.8–47.0)	88.6(88.4–88.9)	0.209 (0.201–0.217)
Yes	52.4(49.8–54.9)	84.1(83.5–84.7)	0.243 (0.239–0.246)	49.2(47.1–51.2)	82.4(82.0–82.8)	0.179 (0.171–0.187)
**Tobacco Consumption**						
No	40.4(40.0–40.8)	91.8(91.7–91.8)	0.287 (0.284–0.290)	46.8(45.3–48.4)	87.4(87.1–87.6)	0.211 (0.203–0.219)
Yes	48.3(47.0–49.7)	88.6(88.3–88.9)	0.296 (0.293–0.300)	46.8(44.6–48.9)	85.5(85.1–85.9)	0.179 (0.172–0.187)
**Region**						
North	39.3(38.6–39.9	92.3(92.2–92.4)	0.299 (0.296–0.303)	45.1(43.0–47.3)	87.5(87.1–87.8)	0.204 (0.196–0.212)
Northeast	51.3(50.2–52.3)	88.0(87.8–88.2)	0.307 (0.303–0.310)	57.8(54.6–60.9)	82.1(81.4–82.7)	0.223 (0.215–0.231)
Central	50.3(48.9–51.7)	92.0(91.8–92.1)	0.286 (0.283–0.290)	60.3(55.4–65.1)	88.1(87.5–88.6)	0.170 (0.162–0.178)
Eastern	38.2(37.2–39.2)	92.7(92.6–92.9)	0.277 (0.274–0.281)	39.4(36.2–42.7)	89.5(89.0–90.0)	0.192 (0.184–0.200)
Western	51.2(49.4–53.0)	91.1(90.9–91.4)	0.286 (0.282–0.289)	56.3(51.4–61.1)	85.9(85.2–86.6)	0.169 (0.161–0.176)
South	32.1(31.3–32.9)	91.5(91.3–91.7)	0.250 (0.246–0.253)	39.8(37.3–42.4)	85.1(84.5–85.7)	0.194 (0.186–0.202)
**Total**	41.1(40.7–41.5)	91.5(91.5–91.6)	0.288 (0.285–0.292)	50.4(49.3–51.6)	85.8(85.6–86.0)	0.199 (0.191–0.207)

The sensitivity of self-reported hypertension was higher among women (48%) and men (56%) belonging to other religions (Christian, Sikh, Jain, etc.) than among their counterparts. In the social caste group, the sensitivity of self-reported hypertension was lowest among women (37%) and men (42%) belonging to the OBC caste, whereas specificity was high among both women and men. Women and men aged 15–49 years in the lowest socioeconomic group had the lowest sensitivity (37% and 37%, respectively) and highest specificity (92% and 90%, respectively) of self-reported hypertension than other socioeconomic groups. The sensitivity among pregnant women was 22.2%, with a low level of agreement between the self-reported and tested hypertension. Furthermore, the sensitivity and specificity among obese respondents were comparatively better than those among their counterparts.

The analysis further suggested that sensitivity increased as the respondent moved from the poorest quintile to the richest quintile for both women and men. The results also showed that sensitivity was higher among women (52%) and men (48%) who consumed alcohol and tobacco. Region-wise analysis showed that the sensitivity of self-reported hypertension was higher among women from the north-eastern region (51%) and lowest among those from the southern region (28%). Similarly, men belonging to the north-east and western regions had a higher sensitivity to self-reported hypertension (51%), and the lowest sensitivity was found among men from the eastern and northern regions.

### 3.3 Respondent characteristics associated with false positivity

The effects of respondents’ characteristics on FP reporting of hypertension based on a multilevel logistic regression model are presented in Table 3. Women aged ≤35 years were selected as the reference group for FP responses based on observations from the previous sections. It was found that as the age of the women increased up to 35 years, the likelihood of FP errors in self-reported hypertension also increased. Similar results were noted for FP responses among women biomedically tested for hypertension but not taking any medication. The results showed that women with a secondary level of education were strongly and significantly associated with more FP errors in self-reported hypertension (adjusted odds ratio [AOR] = 1.27 [taking medication], p<0.01; 1.26 [not taking medication], p<0.01). The likelihood of FP reporting was also higher among women with higher education in both categories of hypertension.

**Table 3:** Multilevel logistic regression modelling for FP and FN responses on hypertension among women and men, NFHS-4, 2015-16.

Background Characteristics	Hypertension, Women age<35 AOR (95% CI)		Hypertension, Women age≥35 AOR (95% CI),		Hypertension Men (15–54) AOR (95% CI)	
	False Positive with medication	False Positive without medication	False Negative with medication	False Negative without medication	False Negative with medication	False negative without medication
**Age**						
15-19®						
20–24	2.41***(2.29,2.53)	2.33***(2.22,2.44)			1.86***(1.68,2.06)	1.98***(1.78,2.2)
25–29	3.15***(3.01,3.32)	3.06***(2.93,3.21)			2.45***(2.22,2.7)	2.64***(2.38,2.93)
30–34	3.31***(3.14,3.48)	3.39***(3.23,3.56)			3.32***(3.01,3.66)	3.59***(3.24,3.98)
35-39®*					4.15***(3.77,4.57)	4.59***(4.15,5.08)
40–44			1.26***(1.23,1.3)	1.28***(1.24,1.32)	4.95***(4.48,5.45)	5.48***(4.94,6.07)
45–49			1.51***(1.47,1.56)	1.53***(1.48,1.57)	5.78***(5.24,6.37)	6.42***(5.79,7.12)
50–54					6.16***(5.56,6.82)	6.81***(6.12,7.58)
**Education**						
No education®						
Primary	1.09***(1.03,1.15)	1.11***(1.06,1.17)	1.02(0.99,1.06)	1.02(0.99,1.06)	1.03(0.96,1.11)	1.04(0.97,1.12)
Secondary	1.27***(1.22,1.33)	1.26***(1.21,1.32)	0.97*(0.94,1)	0.97(0.94,1.01)	1.02(0.96,1.08)	1.01(0.95,1.08)
Higher	1.21***(1.14,1.29)	1.19***(1.13,1.26)	0.84***(0.79,0.89)	0.83***(0.78,0.89)	1.03(0.95,1.11)	1.003(0.93,1.09)
**Current Pregnancy**						
No ®						
Yes	2.32***(2.06,2.61)	2.27***(2.04,2.52)	2.86***(2.19,3.75)	2.90***(2.2,3.82)		
**Body mass index**						
Normal®						
Underweight	0.89***(0.86,0.93)	0.92***(0.88,0.95)	0.73***(0.7,0.76)	0.72***(0.69,0.76)	0.61***(0.57,0.65)	0.59***(0.56,0.64)
Obesity	1.4***(1.34,1.46)	1.45***(1.4,1.51)	1.71***(1.66,1.76)	1.74***(1.7,1.79)	1.93***(1.84,2.02)	1.97***(1.88,2.07)
Not known	0.59***(0.53,0.66)	0.61***(0.55,0.67)	0.21***(0.18,0.25)	0.21***(0.17,0.26)	0.10***(0.07,0.13)	0.10***(0.08,0.14)
**Place of residence**						
Urban®						
Rural	0.96**(0.93,1)	1.02(0.98,1.05)	1.01(0.98,1.04)	1(0.97,1.03)	1.01(0.97,1.06)	1.01(0.96,1.06)
**Religion**						
Hindu®						
Muslim	1.10***(1.05,1.16)	1.15***(1.1,1.19)	1.07***(1.03,1.12)	1.08***(1.04,1.12)	0.92**(0.86,0.99)	0.92**(0.85,0.98)
Others	0.97(0.92,1.02)	0.99(0.94,1.03)	1.04**(1,1.08)	1.05**(1.01,1.09)	1.1***(1.04,1.17)	1.13***(1.06,1.2)
**Caste**						
SC/ST®						
OBC	1.09***(1.05,1.13)	1.07***(1.03,1.11)	0.88***(0.86,0.91)	0.88***(0.85,0.91)	0.86***(0.82,0.91)	0.86***(0.82,0.9)
General	0.87***(0.83,0.91)	0.89***(0.85,0.93)	0.94***(0.91,0.98)	0.94***(0.91,0.97)	0.95*(0.89,1)	0.95*(0.89,1)
Others	0.84***(0.77,0.91)	0.91***(0.84,0.97)	1(0.94,1.06)	0.98(0.92,1.05)	0.88**(0.79,0.98)	0.89**(0.8,0.99)
**Wealth quintile**						
Poorest®						
Poorer	1.25***(1.19,1.33)	1.26***(1.2,1.32)	0.97*(0.93,1)	0.97(0.93,1.01)	1.11***(1.03,1.19)	1.12***(1.04,1.2)
Middle	1.53***(1.45,1.61)	1.49***(1.41,1.56)	0.91***(0.88,0.95)	0.91***(0.87,0.95)	1.17***(1.09,1.26)	1.18***(1.1,1.27)
Richer	1.78***(1.67,1.88)	1.71***(1.62,1.81)	0.89***(0.85,0.93)	0.89***(0.85,0.94)	1.26***(1.17,1.36)	1.27***(1.18,1.37)
Richest	1.70***(1.6,1.82)	1.66***(1.56,1.76)	0.78***(0.74,0.82)	0.77***(0.73,0.81)	1.23***(1.13,1.34)	1.25***(1.14,1.36)
**Alcohol Consumption**						
No®						
Yes	1.01(0.9,1.12)	0.99(0.9,1.09)	1.59***(1.5,1.68)	1.62***(1.53,1.72)	1.32***(1.26,1.37)	1.35***(1.29,1.41)
**Tobacco Consumption**						
No®						
Yes	0.94*(0.88,1.01)	0.96(0.9,1.02)	1(0.96,1.04)	1.01(0.97,1.05)	1(0.96,1.05)	1(0.96,1.04)
Constant	0.01***(0.01,0.01)	0.01***(0.01,0.01)	0.12***(0.12,0.13)	0.12***(0.11,0.12)	0.03***(0.03,0.03)	0.03***(0.02,0.03)
**Random effects (intercept only)**						
σ^2^_PSUs_ (SE)	0.689(0.032)	0.570(0.027)	0.021(0.003)	0.023(0.003)	0.066(0.009)	0.070(0.009)
Intra class correlation (ICC) (PSU)	0.155	0.134	0.006	0.007	0.018	0.018
σ^2^_HHs_ (SE)	0.457(0.006)	0.378(0.025)	0.021(0.020)	0.017(0.022)	0.405(0.045)	0.457(0.047)
Intra class correlation (ICC) (HHs)	0.258	0.224	0.012	0.012	0.125	0.138
Number of observations (n)	459,957	459,957	239,729	239,729	111,821	111,821
Wald chi2(22)	4751.91	5537.07	3633.62	3726.63	4745.07	4779.23
Prob > chi2	0.000	0.000	0.000	0.000	0.000	0.000

Note: ®: reference category; ®*: reference category for FN responses among women aged 35 and above.

Further disaggregated analysis showed that women who were pregnant at the time of the survey had a higher likelihood of FP reporting of hypertension. The AORs for FP responses for women taking and not taking medication were 2.32 (p<0.01) and 2.27 (p<0.01), respectively. This finding is in line with the sensitivity analysis conducted in the previous section, which indicated that the majority of women were unaware of their exact health conditions. Another finding from the study suggests that FP reporting of hypertension was less likely among rural women (AOR = 0.96, p<0.1) than among their urban counterparts in cases where they were receiving medication. The results also showed that FP errors in reporting were more likely among Muslim (AOR = 1.10, p<0.01) than Hindu women. Similar results were obtained for Muslim women in both hypertensive women under medication and those not taking medication.

Women belonging to general (AOR = 0.87, p<0.01) and other social caste groups (AOR = 0.83, p<0.01) were significantly less likely to over-report hypertension. In contrast, the likelihood of FP errors was higher among women belonging to the OBC category. Surprisingly, FP errors in the reporting of hypertension increased with family affluence. Women belonging to the richest (AOR = 0.87; p<0.01), rich (AOR = 0.87; p<0.01), and middle (AOR = 0.87; p<0.01) wealth quintiles were strongly and significantly associated with FP reporting in hypertension. This finding is contradictory to the sensitivity test results, where reporting of hypertension improved from the poorer to the richest wealth groups.

The random part of the multilevel model showed that the variation in FP reporting in hypertension among women aged <35 years was higher at the household level (σ^2^_HHs_ = 0.0.69) than at the community level (σ^2^_PSUs_ = 0.46). Based on intraclass correlation coefficient values, 25.8% and 15.5% of the total variation in FP reporting of self-reported hypertension among women were attributable to differences across communities and households, respectively. Similarly, the ICC values for women aged <35 years under the non-medication category showed that 22% and 13% of the total variation in FP reporting of hypertension were attributable to differences across communities and household levels.

### 3.4 Respondent characteristics associated with false negativity

The evidence of FN or self-underreporting of hypertension presented a unique picture among men and women in India. Women aged 35–49 years and men aged 15–54 years systematically underreported hypertension. The results also showed that specificity decreased with an increase in respondent age. This section explores the respondents’ characteristics regarding FN responses to hypertension in greater detail. The results in Table 3 show that women aged 45–49 years were more likely to underreport hypertension (AOR = 1.51; p<0.01). Similar findings were conspicuous among men, where the magnitude of FN responses increased systematically with age.

The results also showed that place of residence did not have any significant effect on the accurate reporting of the absence of hypertension in both men and women. Muslim women (AOR = 0.84; p<0.01) and women of other religions were significantly associated with FN reporting of hypertension. In contrast, Muslim men were significantly less likely to underreport hypertension (AOR = 0.92; p<0.05). FN reporting of hypertension were significantly less likely among men and women belonging to the OBC (AOR = 1.04; p<0.01) and other social caste groups (AOR = 1.11; p<0.05). Interestingly, FN reporting declined with an increase in women’s educational attainment. However, FN reporting was not significantly associated with men’s educational attainment. Another intriguing observation in this study was the relationship between the wealth index and the pattern of FN reporting of hypertension. It was found that with increasing family wealth, women were less likely to underreport hypertension. This was in contrast with earlier findings related to the FP reporting of hypertension. FN reporting of hypertension among men had a strong positive association with household wealth. This implied that men belonging to the poorest and poorer wealth quintiles had less underreported prevalence of hypertension compared to their counterparts. The analysis also suggested that FN responses to hypertension were positively impacted by lifestyle factors such as obesity and alcohol consumption.

A higher likelihood of FN reporting existed among men (AOR = 1.35; p<0.05) and women (AOR = 1.62; p<0.05) who reportedly consumed alcohol. The analysis also indicated that obese respondents were less likely to report correct responses regarding hypertension. The FN responses to hypertension were more elucidated among men aged 15–54 years (AOR = 1.97; p<0.01) and women aged 35–49 years (AOR = 1.74; p<0.01).

The random part of the multilevel logistic regression model revealed that variation in the underreporting of the presence of hypertension among women was abysmally low at the community (σ^2^_PSUs_ = 0.021) and household (σ^2^_HHs_ = 0.021) levels. The variation in underreporting of the presence of hypertension among men was higher at the household level (σ^2^_HHs_ = 0.41) than at the community level (σ^2^_PSUs_ = 0.07). Based on the ICC values, approximately 1.8% and 45.7% of the total variation in the FN reporting of hypertension among men was attributable to differences across community and household levels, especially among those who were not under any medication. The ICC values for women aged 35–49 years in the non-medication category showed that 0.7% and 1.2% of the total variation in FN reporting on hypertension was attributable to differences across community and household levels.

### 3.5 Robustness check

Sub-sample analyses were performed using two setups to identify consistency in the findings obtained from the full sample. The first sub-sample analysis was conducted using data from the north-eastern states where the prevalence of hypertension was the highest. The second subsample analysis was conducted using data from Uttar Pradesh, which is the most populous state in India and has a larger family size. The results in the supplementary material (Supplementary Table S2 and Table S3) demonstrate that the causality established in the subsample analysis agrees with the findings based on the total sample. The results from the subsample analysis demonstrated that an increase in age, education, wealth status, pregnancy status, and obesity were significant predictors of FP responses among women aged 15–35 years, as was obtained from the full sample analysis. Similarly, among women belonging to the 35–49 years age category, the subsample analysis showed a sustained increase in FN reporting with age. It is evident from the analysis that FN responses have a positive association with pregnancy status, obesity, and lifestyle factors such as alcohol consumption. These significant predictors of FP responses were also observed by Huerta et al. (2009). The results of the robustness check carried out on FP responses on hypertension based on the subsample of men in Uttar Pradesh and north-eastern states (Manipur, Meghalaya, Mizoram, Nagaland, Sikkim, Tripura, Arunachal Pradesh, and Assam) were also in line with the results from the total sample. However, a few differences were also observed between the subsample and full sample analyses. This could be due to regional and contextual factors that give rise to state-level variations.

## 4 Discussion

The present study was an attempt to assess the inconsistency between self-reported and biomedically tested hypertension among men and women in India using NFHS-4 data. This study examined the discrepancy in the prevalence of self-reported hypertension and its estimated prevalence based on biomarker tests performed with consent from eligible women and men in NFHS-4 (2015–16). This study found large inconsistencies between self-reported and biomedically examined results of hypertension in both women and men in India. One could conveniently infer from the study that relying upon self-reporting of hypertension may lead to a significant underestimation of the hypertension burden among men and women. For example, in women and men, self-reporting led to underestimation of hypertension by 20% and 55%, respectively. This may reflect issues of recall bias or actual unawareness of the condition owing to the failure to undertake testing. Similar results have been reported in a number of studies in China (Ning et al., 2016; Xie & Wang, 2020) and India (Bhatia et al., 2021; Onur & Velamuri, 2018; Puri et al., 2020; Shivashankar et al., 2021; Tenkorang et al., 2015).

The use of population-wide data from other nations collected through self-reporting as a tool for policy monitoring or assessment must be done with caution, considering regional disparities, particularly in terms of healthcare access (Goncalves et al., 2018). Low sensitivity may be the result of difficulties or inability to receive health treatments, thus limiting illness awareness. The frequency of visits to the doctor (Lima-costa et al., 2004), education level (Jaddou et al., 2011), and residence in an urban area (Ning et al., 2016) have all been linked to self-reporting accuracy. Furthermore, when retrospective questions concerning chronic conditions are asked, memory bias is always possible (Goncalves et al., 2018). A significant deviation in self-reported hypertension between men and women was noted in this study. An interesting finding from the study is related to the crossover in the pattern of reporting in women of the reproductive age group. This study showed that self-reported hypertension tends to be more valid among women and men residing in urban areas than in rural areas. This might be because the urban areas of India have better economic resources, health care facilities, and accessibility than rural areas. The study also examined the sociodemographic characteristics correlated with underreporting using two indicators: sensitivity and specificity. The findings revealed that women with higher education were less likely to accurately report self-reported hypertension, whereas they were more likely to correctly report the absence of hypertension. Similarly, men with higher education were more likely to accurately report self-reported hypertension and less likely to report the absence of hypertension.

Besides studying the correct reporting of hypertension, a significant section of the study was devoted to analysing the incorrect reporting of hypertension. An elucidative outcome of such exercise is that women aged <35 years were more likely to self-overreport hypertension. This evidence is puzzling and requires in-depth examination through back-check surveys. One reason could be that the majority of women had given birth at least once before reaching 35 years of age (Granger et al., 2001; Magee et al., 1999; Zhang et al., 1997). Several other researchers have indicated the risk of hypertension at the time of pregnancy and the mechanism of its management (Coroyannakis & Khalil, 2019; Magriples et al., 2013; Guedes-Martins et al., 2015; Savitri et al., 2016). It is likely that in large-scale surveys such as the NFHS, over-reporting of hypertension and FP reporting therein are influenced by current pregnancy or recent births in these women. In addition to FP reporting of hypertension, a significant proportion of men and women also underreported the prevalence of hypertension. Presumably, two classes of respondents fell under this category. The first category of respondents was hypertensive with prescribed medication and did not report it at the time of the survey but was found hypertensive when biomedically tested. The second category of respondents included those who were unaware of their current health status. The former responses could be due to the women who were pregnant at the time of the survey and taking presumptive medication against hypertension. The likelihood of FN errors was higher among pregnant women in both categories (with and without medication). Interestingly, among men aged 15–54 years, FN responses to hypertension systematically increased with age. The AOR among men aged 50–54 years was nearly six times higher than that among men aged 15–19 years. Similar results were noted among men who underreported hypertension but reported taking prescribed medication to lower their blood pressure. It is still unclear whether men and women taking medication to control blood pressure incorrectly self-reported hypertension at the time of the survey. This may be because men and women on medication to reduce blood pressure must have self-identified themselves as recovered from hypertension. In contrast, there was a noteworthy proportion of women who self-reported themselves as hypertensive with medication but were not found to be hypertensive when biomedically tested.

In this study, variations in FP and FN reporting of hypertension were highly disproportionate at the community level. Therefore, evidence-based studies on high-risk regions with detailed questions and caveats regarding the reference period could provide segregated information on these inconsistencies. Such variations may be attributed to unobserved contextual factors in participant communities, such as the quality of health education, performance of health systems, accessibility of healthcare resources, and the degree of economic growth within the community. The much higher variance in hypertension prevalence among communities could be due to the contextual effects of community characteristics that were far more apparent in the accuracy of hypertension self-reports, possibly resulting from the fact that hypertension screening accessibility and affordability are much more strongly influenced by community environment.

## 5 Conclusions

Considering the mismatch in the self-reported and biomedically tested results of hypertension in India, it is evident and advisable that all future large-scale surveys should focus on the ascertainment of morbidities through standard tests. Thus, this study recommends a re-examination of the importance of the following three questions from the CAB questionnaire on hypertension status: “*Were you told on two or more different occasions by a doctor or other health professional that you had hypertension or high blood pressure?*”, “*Before this survey, has your blood pressure ever been checked?”,* and “*Are you currently taking prescribed medication to lower your blood pressure?*” and suggests their removal from future NFHS surveys. Biomarkers are considered the gold standard and should be followed, and the removal of unnecessary questions will help smooth the implementation of the survey. Furthermore, the question to assess the respondents’ hypertension status should be asked with a specific reference period. Identified risk groups, such as pregnant women, obese individuals, and those who consume alcohol should be cautiously investigated during the survey. The crossover in the pattern of reporting hypertension among women adds a unique facet to the existing literature. The study ascertained that self-reporting of health status among women is prone to severe over-reporting and under-reporting in reproductive ages. Thus, a series of interventions are needed to increase the outreach of basic health education and the importance of physical examination to citizens, and to promote the use of healthcare to lower the incidence and unawareness of diseases in India. Simultaneously, there is a need to conduct an exploratory study to determine the reasons behind the self-overreporting of hypertension at a disaggregated level.

## 6 Limitation of the study

This study explored a series of new research questions but admittedly has some data limitations. First, the respondents’ recall bias may have impacted self-reported hypertension. Many variables, particularly those related to survey execution and privacy concerns at the time of the survey, were not included in the NFHS datasets. Thus, this aspect of data quality remains unaddressed and presents a serious limitation. Furthermore, the question on hypertension in the NFHS questionnaire was posed without a time frame, making it difficult to determine whether respondents were diagnosed with hypertension recently or a long time ago. Because dietary and cultural practices associated with specific eating habits were not reported in NFHS, this study was not able to distinguish between these causes of hypertension. Furthermore, these variables were not considered in this study.

## Ethics

This study is based on secondary data, is available in public domain for research purpose. Therefore, no ethical approval was required from any institutional review board.

## Funding

This work was supported by the 10.13039/100000865Bill & Melinda Gates Foundation, Seattle, WA [grant # OPP1194597].

## Financial disclosure statement

This paper was written as part of DataQi project of the Population Council funded by 10.13039/100000865Bill & Melinda Gates Foundation (grant **#** OPP1194597).

## Author statement

Shri Kant Singh: Conceptualization; Validation; Investigation; Writing-Review & Editing; Supervision.

Santosh Kumar Sharma: Methodology; Formal Analysis; Writing- Original Draft; Writing-Review & Editing.

Sanjay K Mohanty: Review & Editing; Validation.

Rakesh Mishra: Methodology; Formal Analysis; Writing- Original Draft; Writing-Review & Editing; Validation.

Akash Porwal: Writing - Review & Editing; Validation.

Bal Kishan Gulati: Review & Editing; Validation; Supervision.

## Declaration of competing interest

The authors declare that they have no conflict of interest.

## Data Availability

Data will be made available on request.
