# Supplementary material for: Inconsistency in prevalence of hypertension based on self-reports and use of standard tests: Implications for large scale surveys
Source: SSM Popul Health. 2022 Oct 3;19:101255. doi: 10.1016/j.ssmph.2022.101255 (PMC9547289; doi:10.1016/j.ssmph.2022.101255)
Supplement: Multimedia component 1 [file mmc1.docx]

**Supplementary Tables**

| **Table S1: Prevalence of self-reported and tested measure of hypertension among men and women aged 15-49, NFHS-4 (2015-16)** | | | | | | |
| --- | --- | --- | --- | --- | --- | --- |
|  | **Women** | | | **Men** | | |
|  | **Self-reported** | **Tested** | **Under reported** | **Self-reported** | **Tested** | **Under reported** |
| **Age** |  |  |  |  |  |  |
| 15-24 | 4.6 | 3.6 | -26.4 | 2.9 | 5.2 | 44.4 |
| 25-34 | 9.1 | 8.6 | -5.9 | 5.9 | 13.7 | 57.0 |
| 35-49 | 13.6 | 20.4 | 33.5 | 10.3 | 24.7 | 58.3 |
| **Education** |  |  |  |  |  |  |
| No education | 8.9 | 14.1 | 37.3 | 5.9 | 15.5 | 61.9 |
| Primary | 9.7 | 13.2 | 26.3 | 5.7 | 16.6 | 65.8 |
| Secondary | 8.9 | 9.3 | 4.3 | 6.1 | 13.8 | 56.0 |
| Higher | 9.5 | 7.9 | -19.9 | 8.7 | 16.4 | 47.1 |
| **Current Pregnancy** |  |  |  |  |  |  |
| No | 9.1 | 11.3 | 19.1 |  |  |  |
| Yes | 8.2 | 4.2 | -92.7 |  |  |  |
| **Body mass index** |  |  |  |  |  |  |
| Normal | 8.0 | 9.4 | 15.6 | 6.2 | 14.2 | 56.1 |
| Underweight | 5.2 | 5.7 | 8.7 | 3.2 | 6.7 | 52.4 |
| Obesity | 16.7 | 22.8 | 26.8 | 13.7 | 31.3 | 56.4 |
| Not known | 8.4 | 5.1 | -64.4 | 11.1 | 25.6 | 56.5 |
| **Place of residence** |  |  |  |  |  |  |
| Urban | 11.2 | 12.1 | 7.2 | 7.9 | 16.6 | 52.4 |
| Rural | 8.0 | 10.4 | 23.2 | 5.6 | 13.7 | 59.1 |
| **Religion** |  |  |  |  |  |  |
| Hindu | 9.0 | 10.6 | 15.4 | 6.5 | 14.8 | 56.3 |
| Muslim | 9.1 | 12.1 | 25.4 | 6.2 | 13.1 | 52.4 |
| Others | 10.7 | 13.4 | 20.1 | 6.6 | 18.1 | 63.5 |
| **Caste** |  |  |  |  |  |  |
| SC/ST | 8.0 | 10.4 | 22.8 | 5.7 | 14.5 | 60.8 |
| OBC | 9.8 | 10.5 | 7.3 | 7.2 | 14.5 | 50.5 |
| General | 9.2 | 12.1 | 23.9 | 6.2 | 15.9 | 60.8 |
| Others | 8.9 | 13.6 | 34.4 | 5.5 | 13.4 | 59.0 |
| **Wealth quintile** |  |  |  |  |  |  |
| Poorest | 5.1 | 9.3 | 45.5 | 3.2 | 10.5 | 69.8 |
| Poorer | 7.3 | 9.9 | 25.9 | 4.7 | 11.7 | 59.9 |
| Middle | 9.3 | 10.4 | 11.2 | 6.4 | 14.6 | 56.1 |
| Richer | 11.2 | 12.3 | 9.3 | 7.8 | 17.2 | 54.5 |
| Richest | 12.0 | 12.6 | 5.4 | 8.8 | 18.0 | 50.9 |
| **Alcohol Consumption** |  |  |  |  |  |  |
| No | 9.1 | 10.9 | 16.5 | 5.5 | 12.9 | 57.3 |
| Yes | 8.1 | 18.0 | 54.9 | 8.7 | 19.3 | 54.6 |
| **Tobacco Consumption** |  |  |  |  |  |  |
| No education | 9.1 | 10.8 | 15.7 | 6.7 | 14.3 | 53.4 |
| Yes | 9.4 | 15.3 | 38.4 | 5.6 | 15.8 | 64.7 |
| **Region** |  |  |  |  |  |  |
| North | 9.1 | 10.2 | 10.9 | 6.1 | 13.6 | 55.4 |
| Central | 9.2 | 17.1 | 46.2 | 6.9 | 20.1 | 65.7 |
| Eastern | 4.8 | 10.0 | 51.6 | 2.7 | 13.0 | 79.3 |
| Northeast | 7.4 | 10.3 | 27.8 | 5.6 | 12.4 | 54.9 |
| Western | 6.3 | 11.5 | 44.9 | 4.0 | 16.1 | 75.5 |
| South | 14.2 | 11.7 | -21.4 | 11.0 | 16.9 | 35.0 |
| **Total** | 9.1 | 11.0 | 17.2 | 6.5 | 14.8 | 56.3 |

**Table S2:** **Robust Check: Multilevel logistic regression modelling for FP and FN responses on hypertension among women in Uttar Pradesh, and North-eastern states**

| Background Characteristics | **Uttar Pradesh** | | | | **North Eastern States** | | | |
| --- | --- | --- | --- | --- | --- | --- | --- | --- |
|  | Hypertension OR (95% CI), age<35 | | Hypertension OR (95% CI), age>35 | | Hypertension OR (95% CI), age<35 | | Hypertension OR (95% CI), age>35 | |
|  | False Positive with medication | False Positive without medication | False Negative with medication | False Negative without medication | False Positive with medication | False Positive without medication | False Negative with medication | False Negative without medication |
| **Age** |  |  |  |  |  |  |  |  |
| 15-19 |  |  |  |  |  |  |  |  |
| 20-24 | 3.7***(3.12,4.4) | 3.32***(2.84,3.88) |  |  | 2.1***(1.82,2.43) | 2.09***(1.83,2.38) |  |  |
| 25-29 | 6.26***(5.2,7.53) | 5.73***(4.86,6.76) |  |  | 2.72***(2.37,3.14) | 2.66***(2.34,3.02) |  |  |
| 30-34 | 7.11***(5.85,8.65) | 6.89***(5.79,8.21) |  |  | 2.92***(2.53,3.38) | 3.07***(2.69,3.5) |  |  |
| 35-39 |  |  |  |  |  |  |  |  |
| 40-44 |  |  | 1.34***(1.22,1.48) | 1.38***(1.24,1.53) |  |  | 1.22***(1.13,1.31) | 1.23***(1.14,1.32) |
| 45-49 |  |  | 1.6***(1.44,1.78) | 1.67***(1.48,1.87) |  |  | 1.31***(1.22,1.41) | 1.32***(1.23,1.42) |
| 50-54 |  |  |  |  |  |  |  |  |
| **Education** |  |  |  |  |  |  |  |  |
| No education |  |  |  |  |  |  |  |  |
| Primary | 1.21**(1.01,1.45) | 1.28***(1.09,1.51) | 1.05(0.93,1.19) | 1.06(0.93,1.2) | 1.19*(1,1.43) | 1.21**(1.03,1.42) | 0.84***(0.77,0.92) | 0.84***(0.77,0.92) |
| Secondary | 1.38***(1.19,1.61) | 1.38***(1.2,1.58) | 0.97(0.86,1.08) | 0.97(0.86,1.09) | 1.23***(1.06,1.44) | 1.19**(1.04,1.37) | 0.80***(0.74,0.86) | 0.79***(0.73,0.86) |
| Higher | 1.43***(1.18,1.74) | 1.41***(1.19,1.69) | 0.85(0.7,1.04) | 0.82*(0.66,1.01) | 1.12(0.92,1.37) | 1.09(0.91,1.31) | 0.75***(0.65,0.86) | 0.75***(0.65,0.86) |
| **Current Pregnancy** |  |  |  |  |  |  |  |  |
| No |  |  |  |  |  |  |  |  |
| Yes | 1.88***(1.28,2.77) | 2.07***(1.44,2.96) | 1.16(0.53,2.54) | 1.38(0.61,3.14) | 2.74***(1.97,3.82) | 2.62***(1.93,3.54) | 3.04***(1.82,5.08) | 3.19***(1.9,5.35) |
| **Body mass index** |  |  |  |  |  |  |  |  |
| Normal |  |  |  |  |  |  |  |  |
| Underweight | 0.83***(0.72,0.95) | 0.84***(0.74,0.95) | 0.67***(0.59,0.77) | 0.64***(0.56,0.74) | 0.98(0.86,1.12) | 1.01(0.9,1.14) | 0.87***(0.78,0.96) | 0.88**(0.79,0.98) |
| Obesity | 1.67***(1.44,1.94) | 1.71***(1.5,1.96) | 2.18***(1.93,2.47) | 2.27***(1.99,2.6) | 1.25***(1.11,1.41) | 1.3***(1.16,1.45) | 1.66***(1.55,1.78) | 1.68***(1.57,1.8) |
| Not known | 0.74*(0.51,1.05) | 0.66**(0.47,0.92) | 0.41***(0.24,0.71) | 0.36***(0.2,0.66) | 0.57***(0.42,0.78) | 0.58***(0.44,0.77) | 0.22***(0.15,0.33) | 0.22***(0.15,0.33) |
| **Place of residence** |  |  |  |  |  |  |  |  |
| Urban |  |  |  |  |  |  |  |  |
| Rural | 1.02(0.83,1.26) | 1.17(0.97,1.42) | 1.05(0.94,1.18) | 1.07(0.95,1.2) | 0.90*(0.81,1) | 0.94(0.86,1.04) | 0.96(0.89,1.04) | 0.97(0.9,1.05) |
| **Religion** |  |  |  |  |  |  |  |  |
| Hindu |  |  |  |  |  |  |  |  |
| Muslim | 1.34***(1.14,1.58) | 1.4***(1.2,1.62) | 1.22***(1.09,1.36) | 1.25***(1.11,1.4) | 1.29***(1.08,1.54) | 1.16*(0.98,1.37) | 0.93(0.82,1.05) | 0.92(0.81,1.04) |
| Others | 2.6***(1.3,5.21) | 2.06**(1.07,3.99) | 0.95(0.54,1.67) | 0.93(0.51,1.69) | 1.23***(1.09,1.38) | 1.21***(1.09,1.35) | 0.76***(0.70,0.82) | 0.76***(0.70,0.82) |
| **Caste** |  |  |  |  |  |  |  |  |
| SC/ST |  |  |  |  |  |  |  |  |
| OBC | 1.1(0.95,1.28) | 1.04(0.91,1.18) | 0.98(0.89,1.09) | 0.96(0.86,1.07) | 1.17**(1.005,1.36) | 1.04(0.90,1.20) | 1.01(0.92,1.11) | 1.02(0.93,1.13) |
| General | 1.17*(0.98,1.41) | 1.13(0.96,1.33) | 1.07(0.95,1.22) | 1.05(0.92,1.19) | 1.002(0.85,1.17) | 0.9(0.78,1.04) | 0.85***(0.77,0.94) | 0.86***(0.78,0.96) |
| Others | 0.69(0.25,1.95) | 0.60(0.24,1.54) | 2.07**(1.18,3.65) | 2.03**(1.12,3.68) | 0.96(0.78,1.18) | 1.04(0.87,1.26) | 0.97(0.85,1.11) | 0.98(0.85,1.12) |
| **Wealth quintile** |  |  |  |  |  |  |  |  |
| Poorest |  |  |  |  |  |  |  |  |
| Poorer | 1.55***(1.3,1.85) | 1.51***(1.29,1.77) | 0.97(0.86,1.08) | 0.97(0.86,1.09) | 1.09(0.93,1.29) | 1.13(0.97,1.30) | 1.02(0.93,1.12) | 1.05(0.95,1.15) |
| Middle | 1.67***(1.38,2.02) | 1.7***(1.43,2.01) | 0.85**(0.74,0.97) | 0.84***(0.73,0.96) | 1.5***(1.27,1.78) | 1.52***(1.30,1.77) | 0.9**(0.81,1) | 0.92(0.83,1.02) |
| Richer | 1.97***(1.59,2.42) | 1.83***(1.51,2.2) | 0.81***(0.7,0.94) | 0.81***(0.69,0.94) | 1.73***(1.45,2.08) | 1.72***(1.45,2.03) | 0.83***(0.74,0.93) | 0.85***(0.75,0.96) |
| Richest | 1.71***(1.35,2.18) | 1.65***(1.33,2.05) | 0.79***(0.67,0.94) | 0.78***(0.66,0.93) | 1.82***(1.47,2.25) | 1.68***(1.39,2.05) | 0.78***(0.68,0.91) | 0.79***(0.69,0.92) |
| **Alcohol Consumption** |  |  |  |  |  |  |  |  |
| No |  |  |  |  |  |  |  |  |
| Yes | 0.39(0.06,2.71) | 0.81(0.21,3.16) | 2.35***(1.24,4.47) | 2.28**(1.16,4.5) | 1.31***(1.14,1.5) | 1.22***(1.07,1.38) | 1.29***(1.18,1.4) | 1.31***(1.20,1.43) |
| **Tobacco Consumption** |  |  |  |  |  |  |  |  |
| No |  |  |  |  |  |  |  |  |
| Yes | 0.94(0.71,1.25) | 1.02(0.8,1.31) | 0.85**(0.74,0.97) | 0.84**(0.73,0.97) | 1.26***(1.15,1.38) | 1.22***(1.11,1.33) | 0.80***(0.75,0.86) | 0.80***(0.75,0.86) |
| Constant | 0***(0,0) | 0***(0,0) | 0.07***(0.05,0.1) | 0.06***(0.04,0.09) | 0.01***(0,0.01) | 0.01***(0.01,0.01) | 0.24***(0.21,0.28) | 0.23***(0.2,0.26) |
| **Random effects (intercept only)** |  |  |  |  |  |  |  |  |
| σ^2^_PSUs_ (SE) | 3.460(0.243) | 2.905(189) | 0.278(0.049) | 0.313(0.056) | 0.733(0.068) | 0.644(0.059) | 0.070(0.013) | 0.082(0.015) |
| Intra class correlation (ICC) (PSU) | 0.402 | 0.380 | 0.068 | 0.072 | 0.159 | 0.141 | 0.021 | 0.024 |
| σ^2^_HHs_ (SE) | 1.854(0.273) | 1.456(0.225) | 0.489(0.447) | 0.725(0.513) | 0.589(0.11) | 0.632(0.105) | 0.038(0.075) | 0.035(0.078) |
| Intra class correlation (ICC) (HHs) | 0.618 | 0.570 | 0.189 | 0.240 | 0.287 | 0.279 | 0.032 | 0.034 |
| Number of observations (n) | 67,907 | 67,907 | 29,754 | 29,754 | 63,881 | 63,881 | 34,821 | 34,821 |
| Wald chi2(22) | 661.64 | 788.73 | 218.77 | 197.45 | 568.48 | 630.6 | 593.58 | 596.96 |
| Prob > chi2 | 0.000 | 0.000 | 0.000 | 0.000 | 0.000 | 0.000 | 0.000 | 0.000 |

**Table S3: Robust Check: Multilevel logistic regression modelling for FN responses on hypertension among men aged 15-54 in Uttar Pradesh, and North-eastern states**

| **Background Characteristics** | Hypertension OR (95% CI) -Uttar Pradesh | | Hypertension OR (95% CI) North-eastern states | |
| --- | --- | --- | --- | --- |
|  | False Negative with medication | False negative without medication | False Negative with medication | False negative without medication |
| **Age** |  |  |  |  |
| 15-19 |  |  |  |  |
| 20-24 | 1.72***(1.28,2.31) | 1.79***(1.31,2.45) | 1.83***(1.43,2.33) | 1.86***(1.45,2.38) |
| 25-29 | 1.91***(1.42,2.58) | 2.1***(1.53,2.87) | 2.01***(1.59,2.54) | 2.08***(1.63,2.64) |
| 30-34 | 3.09***(2.3,4.15) | 3.34***(2.45,4.56) | 3.11***(2.47,3.91) | 3.15***(2.49,3.99) |
| 35-39 | 3.92***(2.93,5.23) | 4.37***(3.22,5.92) | 3.59***(2.86,4.51) | 3.71***(2.93,4.68) |
| 40-44 | 4.16***(3.1,5.59) | 4.57***(3.35,6.23) | 4.09***(3.23,5.17) | 4.22***(3.32,5.37) |
| 45-49 | 6.26***(4.67,8.4) | 7.12***(5.22,9.7) | 4.88***(3.86,6.18) | 5.07***(3.99,6.46) |
| 50-54 | 5.09***(3.72,6.96) | 5.69***(4.09,7.91) | 6.12***(4.79,7.82) | 6.45***(5.02,8.28) |
| **Education** |  |  |  |  |
| No education |  |  |  |  |
| Primary | 0.97(0.77,1.23) | 1(0.79,1.27) | 0.75***(0.62,0.91) | 0.74***(0.62,0.9) |
| Secondary | 0.97(0.8,1.18) | 0.98(0.81,1.2) | 0.92(0.78,1.08) | 0.90(0.76,1.06) |
| Higher | 0.98(0.76,1.27) | 0.89(0.68,1.16) | 0.93(0.75,1.14) | 0.91(0.74,1.13) |
| **Body mass index** |  |  |  |  |
| Normal |  |  |  |  |
| Underweight | 0.43***(0.35,0.53) | 0.42***(0.34,0.52) | 0.70***(0.58,0.83) | 0.68***(0.57,0.82) |
| Obesity | 2.25***(1.9,2.66) | 2.39***(2.01,2.84) | 1.86***(1.64,2.1) | 1.89***(1.67,2.14) |
| Not known | 0.34***(0.17,0.66) | 0.32***(0.16,0.66) | 0.06***(0.02,0.13) | 0.06***(0.03,0.13) |
| **Place of residence** |  |  |  |  |
| Urban |  |  |  |  |
| Rural | 0.86(0.72,1.03) | 0.83**(0.68,1) | 0.94(0.82,1.07) | 0.92(0.81,1.05) |
| **Religion** |  |  |  |  |
| Hindu |  |  |  |  |
| Muslim | 0.96(0.79,1.17) | 0.92(0.75,1.13) | 0.92(0.73,1.15) | 0.94(0.75,1.19) |
| Others | 0.55(0.21,1.47) | 0.55(0.2,1.48) | 0.72***(0.63,0.82) | 0.73***(0.64,0.84) |
| **Caste** |  |  |  |  |
| SC/ST |  |  |  |  |
| OBC | 0.99(0.84,1.17) | 1(0.84,1.19) | 0.86*(0.73,1.02) | 0.87(0.74,1.03) |
| General | 0.95(0.77,1.17) | 0.95(0.76,1.18) | 0.86(0.72,1.04) | 0.86(0.71,1.03) |
| Others | 1.61(0.37,7.09) | 1.77(0.39,7.94) | 0.8*(0.63,1.01) | 0.81*(0.64,1.02) |
| **Wealth quintile** |  |  |  |  |
| Poorest |  |  |  |  |
| Poorer | 0.9(0.73,1.1) | 0.88(0.71,1.09) | 0.98(0.82,1.16) | 0.96(0.81,1.14) |
| Middle | 0.96(0.77,1.2) | 0.95(0.75,1.2) | 0.93(0.77,1.11) | 0.9(0.75,1.08) |
| Richer | 1.03(0.81,1.32) | 1.03(0.8,1.32) | 1.09(0.9,1.34) | 1.06(0.87,1.3) |
| Richest | 1.04(0.79,1.36) | 1.05(0.79,1.38) | 1.02(0.8,1.3) | 0.98(0.77,1.26) |
| **Alcohol Consumption** |  |  |  |  |
| No |  |  |  |  |
| Yes | 1.11(0.95,1.31) | 1.16*(0.98,1.36) | 1.23***(1.11,1.38) | 1.25***(1.12,1.39) |
| **Tobacco Consumption** |  |  |  |  |
| No |  |  |  |  |
| Yes | 1.02(0.89,1.18) | 1.02(0.86,1.15) | 0.99(0.90,1.11) | 0.99(0.89,1.10) |
| Constant | 0.03***(0.02,0.05) | 0.03***(0.02,0.04) | 0.07***(0.05,0.10) | 0.07***(0.05,0.10) |
| **Random effects (intercept only)** |  |  |  |  |
| σ^2^_PSUs_ (SE) | 0.285(0.067) | 0.295(0.071) | 0.128(0.033) | 0.122(0.033) |
| Intra class correlation (ICC) (PSU) | 0.069 | 0.070 | 0.033 | 0.031 |
| σ^2^_HHs_ (SE) | 0.552(0.211) | 0.629(0.231) | 0.460(0.135) | 0.496(0.141) |
| Intra class correlation (ICC) (HHs) | 0.203 | 0.219 | 0.15 | 0.158 |
| Number of observations (n) | 13825 | 13,825 | 14509 | 14,509 |
| Wald chi2(22) | 480.98 | 479.14 | 601.68 | 597.41 |
| Prob > chi2 | 0.000 | 0.000 | 0.000 | 0.000 |

Note: North-eastern states include Assam, Sikkim, Manipur, Meghalaya, Nagaland, Mizoram, A
